# Evaluation of Anteroposterior Inclinations of Maxillary Lateral Teeth and Canines Measured on Cephalometric Radiographs in Patients with Skeletal Class I and Class II—A Pilot Study

**DOI:** 10.3390/jcm14124323

**Published:** 2025-06-17

**Authors:** Maciej Warnecki, Marek Nahajowski, Beata Kawala, Joanna Lis, Michał Sarul

**Affiliations:** 1 Independent Researcher, Niemodlińska 63, 45-864 Opole, Poland; 2 Department of Integrated Dentistry, Wrocław Medical University, 50-425 Wrocław, Poland; 3 Department of Dentofacial Orthopedics and Orthodontics, Wrocław Medical University, 50-425 Wrocław, Poland

**Keywords:** humans, retrospective studies, diagnosis, malocclusion, Angle class I, malocclusion, Angle class II, Orthodontics, Orthodontics, corrective

## Abstract

**Background/Objectives**: Routinely, clinicians planning the mechanics of orthodontic treatment for their patients do not take into account the inclinations of canine and lateral teeth. This is due to a lack of solid evidence in the area. Additionally, sound data is lacking on differences between tooth inclinations in patients presenting class II, subdivision 1 and class II, subdivision 2 relationships. This study is meant to investigate this matter. **Methods**: To conduct this study, cephalograms of 83 patients scheduled for orthodontic treatment were retrospectively selected from the records of the Department of Orthodontics at Wroclaw Medical University and analyzed. Patients were divided into three groups (class I, class II subdivision 1, class II subdivision 2). Inclinations of the long axes of canine and lateral teeth were measured in relation to the palatal plane. **Results**: We established that there exist statistically significant differences in inclination for some of the teeth assessed. **Conclusions**: Multiple correlations were found between tooth inclination and some cephalometric measurements, particularly the SNB angle. The canines and upper premolars undergo a natural distal tilt to compensate for the mandible’s retruded position. Clinicians planning treatment for skeletal class II patients should formulate treatment plans involving the distal tipping of lateral teeth with great care, as such patients may already exhibit distal inclinations of the lateral teeth. Patients with skeletal class II, division 2 may present greater demands in terms of proper orthodontic treatment mechanics compared to class II, division 1 patients.

## 1. Introduction

Analysis of the lateral skull (cephalometric) radiograph, together with other measurements collected during physical examination, is one of the key components necessary for establishing a proper diagnosis and treatment plan of the orthodontic patient. Among other measurements, the front teeth inclination and sagittal jaw relationship is assessed during cephalometric analysis. In the literature, the measurement of incisor inclinations and its role in treatment planning has been the subject of numerous analyses [1]. Conversely, the anteroposterior inclinations of lateral teeth and canines have not been routinely assessed to date, to the best of the authors’ knowledge.

This is despite the fact that a common outcome of orthodontic therapy is a change in the position and inclination of the teeth—both anterior and lateral—especially when using non-extraction treatment of patients presenting a class II skeletal relationship. These patients represent a heterogeneous group, which includes division 1 and division 2 characteristics that are different in the aetiology of the malocclusion and transverse characteristics [2,3]. Exploring the matter of lateral teeth and canine inclinations might lead to a better understanding of the possible biological limits of orthodontic treatment of a class II patient.

Thus, it is the belief of the authors that the data obtained during this study may help create more patient-tailored treatment plans by employing accurate mechanics for class II correction. This study aims to compare the anteroposterior inclinations of maxillary canines and lateral teeth among patients with class I, class II/1, and class II/2 malocclusions. Potential correlations with SNA, SNB, ANB, and 1+NA angles are also studied.

## 2. Materials and Methods

This study was approved by the Bioethics Committee at the University of Medical Sciences in Wroclaw, no. KB—415/2022.

The study material consisted of cephalograms and plaster models of 83 patients who reported to the Department of Dentofacial Orthopedics and Orthodontics of Wrocław Medical University.

Inclusion criteria for all the groups were as follows:

- Presence of a clear cephalometric radiograph with long axes of the teeth clearly visible, with no overlapping in the patient file.
- Full permanent upper and lower dentitions present in the study models.
- No history of any craniofacial anomalies.
- Normal vertical relationship (ML-NSL angle 28.0 ± 5.0°).
- Sagittal jaw relationship corresponding with the angle classification of the malocclusion, as found on the plaster models.

Exclusion criteria were as follows:

- Patients presenting class III malocclusion.
- Any history of previous orthodontic treatment.
- Patients with a distinct CR-CO discrepancy that would affect the diagnosis of the malocclusion, as determined during the first consultation by the clinician.

**Figure 1:**
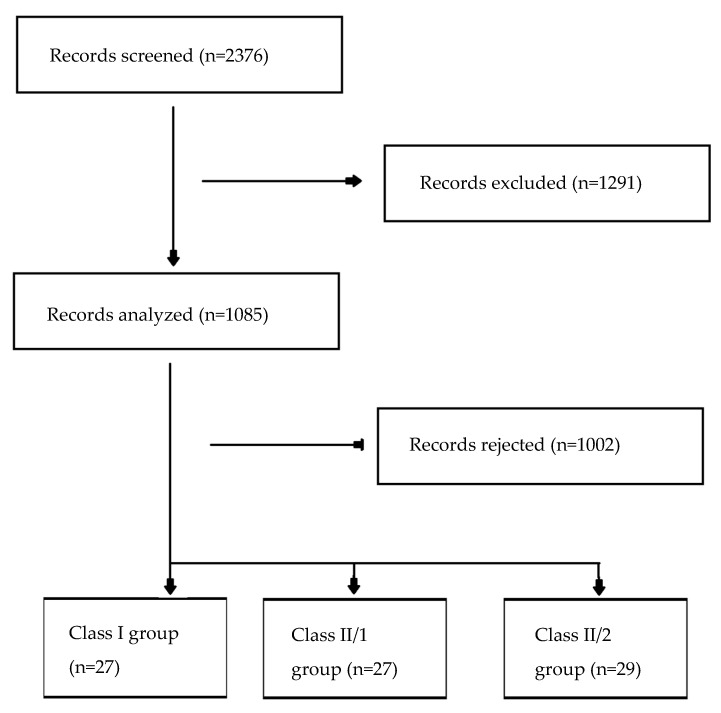
Flowchart presenting the patient files’ analysis: application of inclusion and exclusion criteria and group allocation.

Files of all patients consecutively referred to the Department of Dentofacial Orthopedics and Orthodontics in the years 2019 and 2020 were analyzed. A flow diagram presenting the application of the inclusion and exclusion criteria and group allocation is presented in Figure 1.

Each cephalogram was taken with the same device (Rayscan Alpha Plus, Ray Co., Ltd., Seongnam-si, Gyeonggi-do, Republic of Korea) at 80 kVp, with 11 mAs settings. A standardized protocol for positioning the patient’s head was used. The patients were instructed to look into their eyes in their reflection in a mirror while the image was taken. In this way, a reproducible natural head position was obtained.

A power calculation of the study test was not performed because of the pilot nature of our study.

A Segner–Hasund cephalometric analysis was performed on each of the cephalometric images.

This study included two study groups and one control group:

1. Study group with skeletal class II, division 1 (27 patients: 14 women, 13 men; mean age 21.3 years)—patients presenting class II malocclusion (ANB angle > 4.0°, Wits > 2.0) and having proclined upper incisal incisors.
2. Study group with skeletal class II, division 2 (27 patients: 16 women, 11 men; mean age 22.7 years)—patients presenting class II malocclusion (ANB angle > 4.0°, Wits > 2.0) and having retroclined upper incisal incisors.
3. Control group with skeletal class I (29 patients: 17 women, 12 men; mean age 25.8 years)—patients presenting normal class I skeletal relationship (ANB angle 2.0 ± 2.0°, Wits 0.0 ± 2.0).

All of the patients needed to have a normal vertical relationship (ML-NSL angle 28.0 ± 5.0°). In addition, the following inclusion criteria were applied for the individual groups (all conditions had to be fulfilled simultaneously):

1. Study group with class II, division 1:
  - Overjet ≥ 4 mm (as measured on plaster orthodontic models) measured by calliper.
  - One-half or more of canine and Angle class II bilaterally (as seen on orthodontic plaster models) assessed visually.
  - Inclination of the upper incisors relative to the NA line on the cephalograph at least 1 SD above normal (1+:NA > 25.0°).
2. Study group with class II, division 2:
  - One-half or more of canine and Angle class II bilaterally (as seen on orthodontic plaster models) assessed visually.
  - Inclination of the upper incisors relative to the NA line on the cephalograph at least 1 SD below normal (Angle 1+:NA < 17.0°).
3. Control group with class I:
  - Canine and Angle class I bilaterally (as seen on orthodontic plaster models) assessed visually.

The assessment of tooth inclination was performed according to the methodology already present in the literature [4,5,6,7,8].

Each cephalogram had to be sufficiently sharp and clear so as to delineate the outlines of individual lateral teeth and canines, along with the mesial root of the first molar.

**Figure 2:**
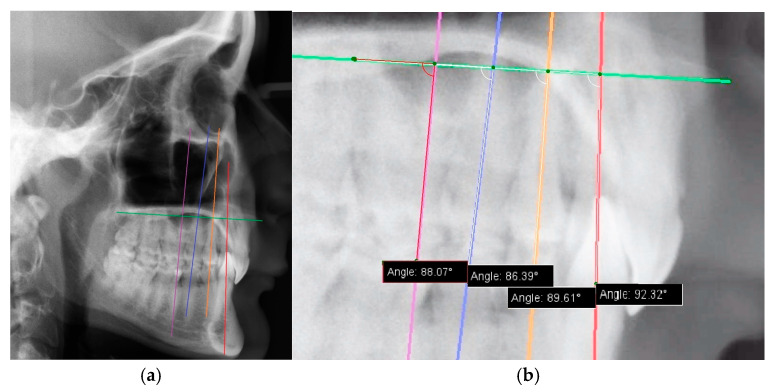
Graphical depiction of the measurement. (**a**) Graphical representation of tooth inclinations and the path of the palatal plane in the study patient. (**b**) An angle is formed and measured between the long axis of the tooth and the palatal plane.

The long axes of the upper canines, the first and second premolar teeth, the first molar, and the palatal plane were marked on the image in a graphics program (Gimp 2.0) [9] (Figure 2a).

To determine the long axis of the first molar, the mesial root was used as an anatomical reference point. The palatal plane was determined as the distance running between the anterior nasal spine and the Pm point.

Two investigators (M.W. and M.N.) were trained in proper palatal plane and tooth long axis marking.

Subsequently, the long axes of the teeth and the path of the palatal plane were marked in all patients independently.

Next, the two investigators were trained in angle measurement with the help of IC Measure 2.0.0.086 software [10] (Figure 2b), and the tooth inclinations (measured as the angle between the palatal plane and the long axis of the tooth) were obtained. Inclination measurements were also taken twice, by each investigator separately, in order to later determine the agreement of measurements between investigators.

In addition, Researcher 1 performed all the markings and measurements again after two weeks to confirm the reliability and repeatability of the results obtained.

Inclinations of the upper canine (3-PP) and first premolar (4-PP), second premolar (5-PP), and first molar (6-PP) were obtained by measuring the angle between the long axis of the respective tooth and the line representing the palatal plane.

Next, the values were entered into STATISTICA v.13.3 software (TIBCO Software Inc., Palo Alto, CA, USA) to perform statistical calculations.

The following calculations were performed on the collected data:

- Intraclass correlation coefficient (ICC) between measurements taken by Researcher 1 (Measurement 1) and Researcher 2, to assess inter-rater reliability.
- Intraclass correlation coefficient (ICC) between two sets of measurements taken by Researcher 1 (Measurement 1 and Measurement 2), to assess intra-rater reliability over time.
- Pearson’s correlation coefficient (parametric) and Spearman’s rank correlation (non-parametric) between measurements of 3-PP, 4-PP, 5-PP, and 6-PP and the angular values SNA, SNB, ANB, and 1+:NA, as described in Segner–Hasund’s cephalometric analysis [11].
- Analysis of variance (Kruskal–Wallis ANOVA test) to assess the statistical significance of differences in the 3-PP, 4-PP, 5-PP, and 6-PP measurements between groups.

The following null hypotheses were formulated:

- There are no statistically significant differences in lateral tooth inclinations among patients with different skeletal classes (class I, class II/1, class II/2).
- There is no statistically significant correlation between lateral tooth inclinations and the angles SNA, SNB, ANB, and 1+:NA in the patient groups included in this study.

## 3. Results

### 3.1. Differences in Tooth Inclination Between the Groups

**Figure 3:**
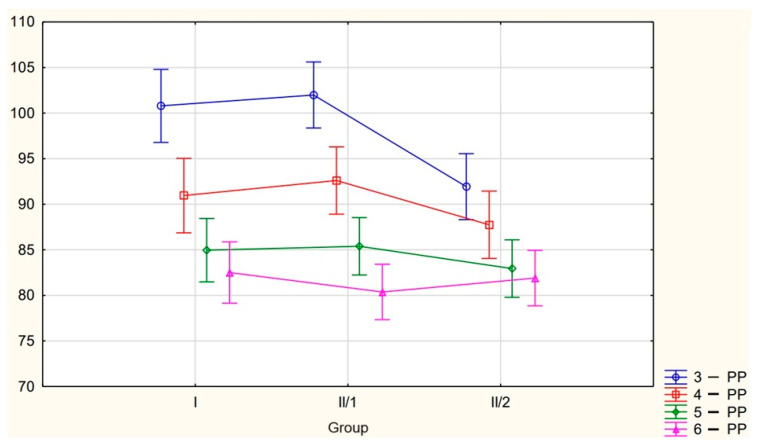
Graphical representation of the differences in tooth inclination between the groups. Vertical bars represent 95% confidence intervals (CIs). The vertical axis represents the angular value.

The graphical representation of the differences between the groups in tooth inclination is presented in Figure 3.

### 3.2. Measurement Reliability

#### 3.2.1. Inter-Examiner Reliability

For teeth 3, 4, and 5, an inter-examiner agreement coefficient above 0.9 was obtained. This indicates a very good reliability of the measurements for these teeth. For the measurements of the first molar, a score above 0.75 was obtained. This indicates a good reliability of these measurements [12].

#### 3.2.2. Intra-Examiner Reliability

For teeth 3, 4, 5, and 6, an agreement coefficient result of more than 0.9 was obtained. This indicates a very high agreement between measurements in the same examiner over time.

### 3.3. Correlation Coefficient Calculation Between Tooth Inclinations and Cephalometric Measurements

#### 3.3.1. Pearson’s (Parametric) Correlation Coefficient

The results of Pearson’s (parametric) correlation coefficient between tooth inclinations and the SNA, SNB, ANB, and 1+:NA angles are presented in Table 1.

#### 3.3.2. Spearman’s (Non-Parametric) Correlation Coefficient

The results of Pearson’s (parametric) correlation coefficient between tooth inclination and the SNA, SNB, ANB, and 1+:NA angles are presented in Table 2.

### 3.4. Comparison of Tooth Inclination Across Skeletal Classes Relationship

**Figure 4:**
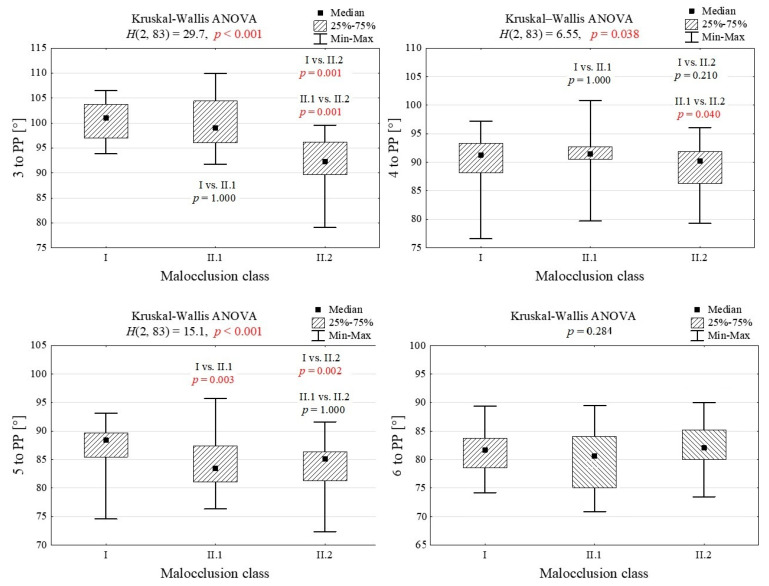
Graphical representation of the analysis of variance by Kruskal–Wallis ANOVA for tooth axes in the between-group comparisons. Values that are statistically significant are marked in red (*p* < 0.05).

A graphical representation of the comparison of tooth inclination across skeletal classes relationship by means of the Kruskal–Wallis ANOVA test is shown in Figure 4.

## 4. Discussion

The purpose of the current study was to determine whether there are differences in the inclinations of the lateral teeth and maxillary canines between patients presenting skeletal class I, skeletal class II division 1, and class II division 2 malocclusions.

Additionally, correlations between the inclinations of the teeth and the SNA, SNB, ANB, and 1+:NA angles were also studied. Differences for some of the inclinations studied were confirmed. This was most pronounced between group II/2 and the other groups. The presence of some correlations between tooth inclination and the studied cephalometric angles was confirmed.

It is the authors’ opinion that those relationships that were statistically confirmed by both Pearson’s (parametric) and Spearman’s (non-parametric) correlation tests deserve particular attention and description. This is due to the fact that observed relationships that are confirmed with both of these statistical tests can potentially be generalized with greater confidence to the entire patient population, and it is less likely that the results obtained are the result of chance.

For the SNA angle in both methods of correlation assessment, a positive relationship was found with the 5-PP angle.

For the SNB angle in both methods of correlation assessment, a positive relationship was found with the 3-PP, 4-PP, and 5-PP measurements. This may suggest that the lateral teeth undergo a natural distal tilt in order to compensate for the distal position of the mandible and that this compensation is linear (the more retruded the mandible, the more distally inclined the canines and upper premolars). Clinically, this information can be used in patients with mandibular retrognathia, where the treatment goal involves orthodontic decompensation of the malocclusion (as, for example, preparation for orthognathic surgery). In such a case, the treatment aims should include the correction of excessive distal inclinations of the upper canines and premolars, for example, with class III elastics.

For the 1+:NA angle, a positive correlation was found with the 3-PP and 4-PP angles in both methods of correlation assessment. The strength of this correlation was particularly evident for the canine (>0.8). This may suggest that in the case of patients with excessively proclined upper incisors, where the treatment objectives include retroclination of the upper incisors, the goal of treatment should also include correction of excessive mesial inclinations of the canine and first upper premolar. This may require more sophisticated orthodontic mechanics and the use of additional means for anchorage control, such as skeletal anchorage (TISAD).

The group that showed the greatest number of statistically significant differences in tooth inclinations compared to the other groups was class II, division 2 (II/2). In this case, statistically significant differences were found for the canine (3-PP) compared to the control and the II/1 group. For the first premolar (4-PP), a statistically significant difference was found compared to the II/1 group. For the second premolar, statistically significant differences were found between the control and both study groups.

It should be noted, however, that the ranges of 25–75% of the measurement values for both upper premolars were relatively close to each other between the groups, and the upper and lower limits of the value ranges were wide. This indicates significant variation in the measurements studied.

For the first molar, no statistically significant correlations between the groups were found.

This is in line with the observations of most authors [7,12,13], whereas, in one of the analyzed papers, the authors found a statistically significant relationship between a patient’s skeletal class and mandibular position and the inclinations of the first upper molar [6].

The above indicates that clinicians planning treatment for patients with class II, division 1 and division 2 should create treatment plans involving distal tipping of the lateral teeth (such as in the total arch distalization technique) with great care. This is due to the fact that many of these patients may already show compensatory distal tipping of the lateral teeth at the start of the treatment. Where such a correlation has not been demonstrated (such as for the first molar), it is very likely that the tooth inclination will already be close to ideal (as presented in the skeletal class I control group) prior to the treatment.

Given the above, clinicians should consider a treatment plan that includes extractions in the upper arch in certain cases.

It is also important to note the greater distal inclination of the canine and first premolar in patients with class II, division 2 compared to division 1. This may suggest that treatment of patients with class II, division 2 malocclusion will require significantly greater distal movement of the canine root and first premolar compared to patients with class II, division 1. This creates challenges for the proper biomechanics of orthodontic treatment, possibly necessitating the use of a temporary skeletal intraoral device (TISAD) by the clinician in such cases.

Considering the above-described correlations and the resulting clinical implications, the authors suggest that the assessment of the inclinations of the maxillary lateral teeth and canines should become part of routine diagnostics and orthodontic treatment planning. Modern radiographs offer sufficient quality to accurately determine the axes of the lateral teeth and canines—assuming that the radiograph is taken correctly.

The analysis of lateral tooth inclinations in relation to the vertical configuration of the patient’s jaws is a further direction worth investigating, as there are reports in the literature of the presence of such relationships in relation to the first upper molars [6,8].

It is, however, important to highlight the limitations of our study. A power calculation of the test was not performed. The size of the study groups was based on convenience-based sampling. This was due to the pilot nature of our work and the fact that group sizes were significantly limited because of strict inclusion criteria (especially the need for a clear cephalometric radiograph with long axes of the teeth clearly visible, with no overlapping). Additionally, the measurement method was not compared against any previously published protocol. This was due to significant heterogeneity in terms of measurement method in the papers concerning tooth long-axis measurement.

Sexual dimorphism, which can affect the results, was not studied—notwithstanding the similar proportion of men and women in each group, which should minimize the risk of any dimorphism affecting the differences between groups. However, future studies could incorporate sex-specific analysis to confirm this assumption.

Because of the assessment of tooth inclinations in the 2D image, it was not possible to precisely determine the position of the roots of the teeth in three dimensions. This represents a limitation of our work.

The possible effect of age differences between the study groups on tooth inclinations was also not analyzed.

Only normodivergent patients were studied, so the relationships described cannot be directly translated to patients presenting other vertical patterns than those studied (low- and high-angle patients).

Given the important findings present in our paper, it seems appropriate to conduct further studies to assess whether similar relationships are present in more clinical scenarios (such as patients with different vertical patterns and class III patients).

## 5. Conclusions

- The canines and upper premolars undergo a natural distal tilt to compensate for the distal position of the mandible, and this compensation is linear.
- Orthodontists planning compensatory treatment of patients with skeletal class II should formulate treatment plans, including distal tipping of the lateral teeth (such as in the total arch distalization technique) with great care, as these patients may already show distal inclinations of the lateral teeth at the beginning of treatment.
- Patients with skeletal class II, division 2 may present greater challenges in terms of correct treatment biomechanics compared to class II, division 1 patients.
- Further studies are warranted to verify the presented patterns on a wider study group and, potentially, on other patient groups, such as skeletal class III patients.

## Figures and Tables

**Table 1:** Results of parametric correlation coefficient calculations. Statistically significant results are marked with an asterisk (*p* < 0.05).

	SNA	SNB	ANB	1+:NA
3-PP	0.3675 *	0.5501 *	−0.2047	0.8118 *
4-PP	0.4390 *	0.5022 *	0.0277	0.5150 *
5-PP	0.5292 *	0.5794 *	0.0791	0.3261
6-PP	0.3638 *	0.3410	0.1548	0.0172

**Table 2:** Results of non-parametric correlation coefficient calculations. Statistically significant results are marked with an asterisk (*p* < 0.05).

	SNA	SNB	ANB	1+:NA
3-PP	0.2708	0.490 *	−0.210	0.838 *
4-PP	0.3061	0.433 *	−0.005	0.5822 *
5-PP	0.4070 *	0.4651 *	0.1115 *	0.2727
6-PP	0.3521	0.3829 *	0.1889	−0.0054

## Data Availability

All data generated and analyzed during this study are available from the corresponding author on reasonable request.
